# Cost-effective, open-source light shutters with Arduino control

**DOI:** 10.1016/j.ohx.2024.e00548

**Published:** 2024-06-27

**Authors:** Mathias S. Fischer, Martin C. Fischer

**Affiliations:** North Carolina State University, Raleigh, NC, USA; Department of Chemistry, Duke University, NC, USA

**Keywords:** Optical shutter, Laser safety, Microscopy, Arduino, Open hardware

## Abstract

In optical experiments, shutters are devices that open or close a path of light. They are often used to limit the duration of light exposure onto a target or onto a detector to reduce possible light-induced damage. Many commercial shutters are available for different applications – some provide very fast opening and closing times, some can handle large optical powers, and others allow for fail-safe operation. Many of these devices are costly and offer limited control options. Here we provide an open-source design for a low-cost, general purpose shutter system based on ubiquitous actuators (servo motors or solenoids) that are connected to an Arduino-based controller. Several shutters can be controlled by one controller, further reducing system cost. The state of the shutters can be controlled via a display built into the controller, by serial commands via USB, or by electrical control lines. The use of a microcontroller makes the shutter controller adaptable – only control options that are used need to be included, and the design accommodates a selection of display and actuator options. We provide designs for all required components, including 3D print files for the actuator holders and cases, the Arduino code, libraries for serial communication (C and python), and example graphical user interfaces for testing.

## Highlights

• Shutters can control light paths in microscopy and spectroscopy applications.
• Low-cost shutters can utilize actuators like servo motors or solenoids.
• An Arduino controller provides flexible interface options.

## Graphical abstract

**Figure:**
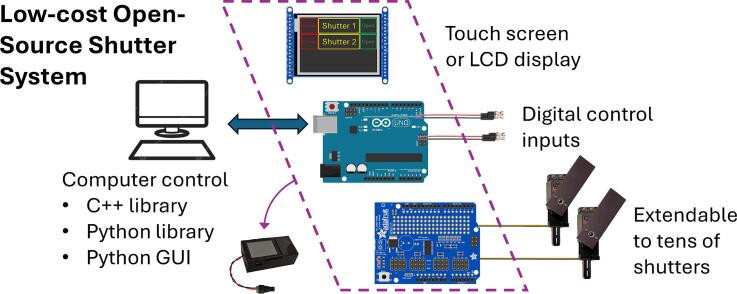

**Specifications table:** 

Hardware name	*Cost-effective, open-source light shutters with Arduino control*
Subject area	General
Hardware type	Other [Laser physics and optical imaging]
Closest commercial analog	*A variety of special-purpose shutters and controllers.*
Open source license	*CERN-OHL-W-2.0 (CERN weakly reciprocal v2)*
Cost of hardware	*4 shutters with controller: ∼$140*
Source file repository	*Zenodo data link:**https://doi.org/10.5281/zenodo.10828203*

## 1 Hardware in context

In many studies that involve a light source, light exposure is intermittent with “on” and “off” periods. Switching could be required for safety reasons (turning the light off while observing the sample by eye), to limit the amount of deposited energy, to switch between different light paths, or to observe a time-dependent response. Repeatedly turning the light source on or off is often too slow (as with halogen lamps) or detrimental to the source (as with some mercury lamps). Mechanical shutters provide a convenient route to control the exposure window by inserting a mechanical block into the light path.

Many designs are in use for the type of mechanical blade, the way the blade is moved into and out of the light path, and the means of controlling the open/closed state [1], [2]. Common blade designs are irises (diaphragms) that open/close radially, or rigid blades that sweep across the path. The blades can block the light by absorbing it (e.g., absorption on dark blades) or by redirecting it (e.g., scattering or reflection on bare metallic blades). The most common options for moving the blades are to attach them to solenoids [3], [4] or to rotary motors [5]. Because of the inertia in mechanical motion, there is a tradeoff between the size of the obstructed light path and the time required to open/close it (and the frequency of opening/closing cycles). In some applications, the size of the light path can be reduced by focusing and placing the shutter at the focal position, thus shortening the opening and closing times. The use of a continuously rotating shutter wheel (chopper, e.g. [6], [7]) can offer vastly increased cycle frequencies but is restricted to cases where opening and closing occurs at regular intervals. Our design is not aimed at high-speed applications, for which commercial devices are superior. Instead, we provide a low-cost, open hardware, general purpose design, which can shutter light at moderate speeds (well within 50 ms). In our lab, we use this system to switch several laser paths in a spectroscopy experiment and to block the laser for background measurements, but we are convinced that our design will prove useful for many other applications. For example, this system is suited for use in a microscope to turn the illumination off when the acquisition is stopped or to switch the light paths for different experimental conditions.

Here we provide a brief overview of the available commercial shutters, which cover different applications areas. Devices with very fast opening/closing times and low latencies are available from Vincent Associates [8] or Thorlabs [9], but a single shutter with the associated driver electronics generally costs in excess of a thousand US dollars. Lower-cost devices are available that sacrifice some performance, mostly opening/closing speeds, or control options. Solenoid-based shutters are available from Brandstrom Instruments [10], EOPC [11], DACO Instruments [12], and KENDRION [13] with a wide range of blade options; Picard Instruments [14] offers a stepper-motor based shutter. Some of these devices are available with controllers and/or a programming interface. Radiant Dyes [15] offers a device based on a servo motor and a controller that opens/closes the shutter via manual switches, digital inputs, or a serial communications port. However, all these lower-cost devices still cost several hundred dollars to operate a single shutter and configuration and control options are very limited.

Many do-it-yourself (DIY) shutter designs have been published in the scientific literature. They vary greatly in performance, cost, and ease of assembly; few provide easy-to-replicate build instructions. Early designs relied on magnetic coils, relays, or solenoids [1], [3], [4], [16], DC motors [5], [17], or loudspeakers [18]. Faster blade motion with lower timing jitter can be achieved with voice coils extracted from hard disk drives [19], [20]; however, this design requires the sacrifice and disassembly of a hard disk drive and custom mounting of the actuator, posing challenges with sourcing and sustainability. Fast switching can also be achieved with a piezo cantilever design [21], [22], [23], but this generally requires a high-voltage drive signal. A simple servo-based shutter has been demonstrated [24] as part of a (only partially completed) spectrometer design, and in a quantum optics lab [25], but for neither demonstration were we able to locate details on the implementation or design files for replication. Of these DIY designs, the 3D printed, DC motor-based design [5] contains part files and electronic design files that aid in replication; for the voice coil design instructional material and videos [26], [27] are available.

**Fig. 1:**
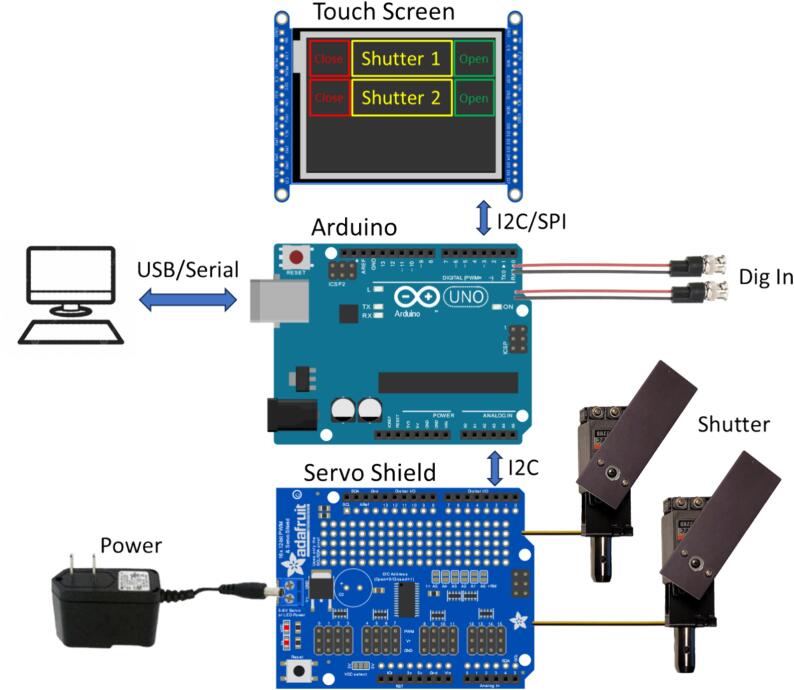
Schematic overview of the shutter system with servo motors and touch screen display.

Here we describe a simple, easy-to-build, low-cost, and open-source shutter with two choices of actuators. The first actuator choice is an RC servo with a rotating blade that can block fairly large beam paths, see Fig. 1 (blade sizes are adaptable up the several centimeters). This actuator’s operation is quiet but exhibits tens of ms timing jitter. The second actuator choice is a solenoid with a blade that moves linearly in and out of the beam path. This design is faster, has a lower timing uncertainty (opening/closing times of sub-10 ms and on/off cycle rates of 10 Hz range are achievable), and provides fail-safe operation since the spring-loaded actuator closes the shutter during power loss. However, the beam size is limited to the linear throw of the solenoid and operation is considerably louder. Both designs use the same Arduino-based controller that offers a wide range of configuration options. Several shutters can be controlled with one controller – we tested the design with four shutters, but extensions to more shutters are straightforward. The shutter controller can be configured to receive input from a display (LCD with push buttons or a touch screen display), from hardware digital control inputs, or through USB serial communication. We provide the control software (Arduino code, a C or python-based library for serial communication, and a python GUI) and the mechanical designs for the shutters and controller enclosures.

## 2 Hardware description

### 2.1 Overall implementation and design

The shutter system consists of the mechanical part and the control electronics. The mechanical shutter is based on a commercial actuator (either an RC servo or a solenoid) mounted in a 3D-printed holder. A black anodized aluminum blade is mounted on the actuator (the rotating horn on the servo or the moving plunger in the solenoid) to block the light path for a given actuator position. The associated control electronics is based on an Arduino Uno with a servo/motor shield for controlling several actuators and a display shield for displaying and switching the state of the shutters. Control of the position of the shutters can occur through user control on the display, serial communication, or TTL-compatible digital control inputs. The provided enclosures and the default hard/software configuration is designed for up to 4 channels, but the design can accommodate tens of shutters with trivial modifications.

### 2.2 Shutter mechanics

The blocking action of the shutter is achieved by rotating or moving an opaque blade into the light path. Here, the blade is made from black anodized aluminum, which absorbs most of the light, exhibits some scattering, but transmits no light. The metallic blade dissipates moderate amounts of absorbed light as heat to the surrounding air. For high-intensity beams, care must be taken not to overheat the actuator and gradual bleaching of the dye used in the anodization process can be expected. For such cases, a mirror may be mounted on the blade to redirect, rather than absorb the light.

**Fig. 2:**
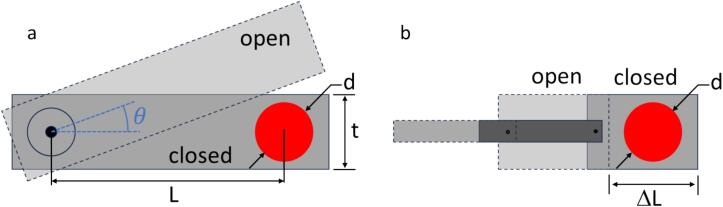
Schematic of the shutter blades for the servo motor design (a) and the solenoid design (b). The red circle indicates the light beam. (For interpretation of the references to color in this figure legend, the reader is referred to the web version of this article.)

For the servo motor design (Fig. 2a), the speed at which the shutter opens/closes the light path depends on the rotational speed of the servo, r (measured in degrees/s), and the required angle of the blade to traverse the light, θ. The angle θ, in turn, depends on the diameter of the light path, d, and its distance, L*,* from the servo axis. If d≪L, the opening and closing time τ can be approximated as

(1) $$\tau =\frac{360^{{}^{\circ}}}{r}\frac{d}{2\pi L}$$

where we assumed a uniform beam and neglected acceleration effects of the servo. Hence, a faster servo, a longer distance between light path and servo axis, and a small light path diameter (maybe even by focusing through a lens) decreases the opening/closing times. For a light beam of a few mm in diameter, a convenient L of a few cm, and typical servo speeds (60°/100 ms), opening and closing times are on the order of several ms. The time delay between an open/close request and the actual light increase/decrease is determined by the width and position of the blade, t, the limited acceleration of the servo, and the delay of the control electronics. Even for a minimal blade width (t=d) the frequency of the servo control signal limits the response to delays and timing jitter of several tens of ms (example performance data are given in the Validation and characterization section below).

For the solenoid design (Fig. 2b), the speed at which the shutter opens/closes the light path also depends on the blade speed. In contrast to servo motors, which have a well-specified rotational speed, the plunger velocity in most solenoids is not specified, highly nonlinear, strongly dependent on what is attached to it (the blade in our case), and often different for opening and closing. Hence, the transit times need to be experimentally determined for each design (see the Validation and characterization section below). The maximum light beam diameter d that can be accommodated with a linear solenoid is limited by the maximum throw ΔL of the plunger.

### 2.3 Controller

The controller for our shutter system is based on an Arduino Uno. The Arduino board can be powered through USB or via an external power supply. To drive the actuators, we utilize dedicated, external Arduino shields (a pulse-width modulation (PWM) board for servos or a motor shield for solenoids) for several reasons:

1. The current that the Arduino 5 V pin can supply is quite limited (800 mA if using the power input, even less if connected to an unpowered USB hub), whereas a single, medium-sized servo or solenoid can already temporarily draw several hundred mA. Hence, we opted for shields with a separate power supply.
2. For servo motor actuators, even though the PWM signals that control the servo motors can be created directly by the Arduino’s digital outputs, controlling several servos requires careful sharing of the Arduino’s resources (especially timers). Hence, using a dedicated, external PWM board significantly simplifies the programming.
3. For solenoid actuators, the drive electronics needs to be able to deliver enough current into the inductive load of the solenoid and be tolerant to the inductive voltage spike when turning the current off. Rather than assembling the drive circuitry from discrete components we opted to utilize a ready-made motor shield for simplicity.

An optional display board serves to display the status of the shutters and to provide a way for user input. The user can also control the shutters via serial commands over USB or via external TTL control inputs monitored by the Arduino. The configuration of the shutters (such as open/close positions, labels, and control port mapping) is stored in the Arduino’s electrically erasable programmable read-only memory (EEPROM). The Arduino, shields, and input BNC connectors are housed within a 3D printed enclosure. Below we describe each of these components in more detail.

#### 2.3.1 Servo shield (used for servo motor actuators)

The servo shield is a 16-channel PWM shield with an Inter-Integrated Circuit (I2C) interface (Adafruit Product ID: 1411). For servo position updates, commands are sent from the Arduino to the shield over the I2C bus. In between updates, the shield holds the servo positions and does not require Arduino involvement. Several shields can be stacked if more than 16 channels are required (though some changes in case design and software would be required). If a display shield is used on top of the servo shield, the required headers limit the space for servo connectors (see build instructions). The Arduino is not able to supply enough power for the use of several servos simultaneously, so the servo shield utilizes a separate 5 V power supply. To provide enough peak current for the simultaneous movement of several servos, place for a storage capacitor is provided on the shield circuit board – its capacity should be matched to the expected number of servos utilized.

#### 2.3.2 Motor shield (used for solenoid actuators)

The motor shield is a 4-channel motor driver shield with an I2C interface (Adafruit Product ID: 1438). Each motor channel can drive one solenoid coil. For actuator position updates, commands are sent from the Arduino to the shield over the I2C bus. In contrast to the servos, our solenoids have only two states: extended (drive current holds the shutter open) and retracted (no current, shutter closed via the built-in spring). In between updates, the shield holds the solenoid state and does not require Arduino involvement. Several shields can be stacked if more than 4 channels are required (though some changes in case design and software would be required). The Arduino is not able to supply enough power for the use of solenoids, so the motor shield also utilizes a separate 5 V power supply.

#### 2.3.3 Display shield

If the use of a display shield is desired, we provide two options: an LCD screen with buttons or a touch screen.

The liquid–crystal display (LCD) shield (Adafruit Product ID: 772) contains a 16x2 character LCD and push buttons. On the display, the first line displays the shutter name, the second line the shutter status (open, closed, or inactive). The up/down buttons cycle through the selected shutter, the left/right buttons change the state (left for close, right for open, an optional timeout sets the shutter to an inactive state).

The touchscreen shield (Adafruit Product ID: 1947) contains a 2.8″, 240 × 320 pixel thin-film-transistor (TFT) LCD with capacitive touch sensing. The display portion uses the Arduino’s Serial Peripheral Interface (SPI) bus, the touch sensing the I2C bus. Each line displays the shutter label in the center, and touch areas (“buttons”) for opening and closing the shutter on either side.

#### 2.3.4 Digital input

To control the shutters with an electrical signal, we implemented an interrupt-based monitoring routine of some of the Arduino digital input ports. The ports are configurable as 0–5 V (TTL) inputs with (optional) input pull-up resistors that also allow the use of simple mechanical single-pole switches. The current case and software allow for four control lines, but this can be easily extended. Each shutter can be mapped to any (or no) control line and each control line can control several shutters. Note that if the inputs are used as an interlock signal for laser safety applications, the spring-loaded solenoids close the shutter in case of a power failure while the servo motors keep their last position and hence do not provide a fail-safe mechanism.

#### 2.3.5 Serial communication

Serial communication via the Arduino’s USB port allows for programming of the shutter parameters, controlling the shutters, and checking the shutter status. Communication is done by sending three-character ASCII commands (with following command parameters, if applicable). A list of commands is provided in Table1. The command format and example responses for the serial commands are listed in Table2.

**Table 1:** List of serial commands.

Command	Description
*IDN?	Gets ID_STRING
GTI	Gets elapsed time in ms
GND	Gets the number of defined shutters
GSTx	Gets the state of shutter number x
GDLx	Gets the label of shutter number x
GTDx	Gets the stored transit delay of shutter number x in ms
GPRx	Gets all the stored parameters for shutter number x
SSTx	Sets the state of shutter number x
SSPx	Sets the current actuator position of shutter number x
SPRx	Sets all the parameters stored for shutter number x
CLR	Clear the shutter parameters
SAV	Save the current shutter parameters to EEPROM

**Table 2:** Format and example responses for the serial commands.

Command	Example responses from the Arduino
*IDN?	“Arduino Uno Shutter 4.0”
GTI	“TI = 434335”
GND	“ND = 3”
GST0	“ST = 1” Note: 0 −> closed, 1 −> open, 2 −> manual position set, −1 −> inactive
GDL1	“DL = ShutterLabel2”
GTD0	“TD = 500”
GPR1	“PR1,11,2,110,130,800, ShutterLabel2”. Format: PRx,shieldChannel,digInputChannel,posOpen,posClosed,transitDelay_ms,Label
SST0,1	Opens shutter number 0 Returns “OK” if successful, an error otherwise. Note: 0 −> close, 1 −> open
SSP0,200	Sets the actuator number 0 to position 200. Returns “OK” if successful, an error otherwise.
SPR1,11,2,110,130,800,ShutterLabel2	Sets the parameters for actuator number 1. Returns “OK” if successful, an error otherwise. Format: see GPRx Note: if x = −1 then a new shutter is added after the existing shutters
CLR	Returns “OK”
SAV	Saves only if at least one shutter is defined. Returns “OK” if successful, an error otherwise.

Additional notes:

• For the communication to the Arduino, line termination is a line feed (‘\n’, LF, 0x0A) by default, but can be changed to a carriage return (CR, ‘\r’, 0x0D). The response from the Arduino is the standard CR/LF (“\r\n”).
• For commands that address a specific shutter (e.g. GSTx) the shutter number x has a zero-based index (0->first shutter, 1->second shutter). The same applies to the input control lines.
• Another SPR example: “SPR-1,3,-1,255,315,400,Name1” adds a new shutter labelled “Name1” after the existing shutters. The new shutter uses the shield channel 3, is not controlled by input lines, and has an open/close position of 255/315, and a transit delay of 400 ms.
• For the motor shield, the “actuator position” determines the average voltage applied to the solenoid coil: 0 means no voltage (closed shutter), 255 is the maximum voltage (opened shutter).

#### 2.3.6 Parameter storage

Each shutter is associated with several parameters:

• “shieldChannel” is the assigned hardware channel number of the servo or motor shield. Range: 0 to 15 for the servo shield, 0 to 3 for the motor shield.
• “digInputChannel” is the input control line that controls the shutter state. Range: 0 to 3 and −1 (not controlled).
• “posOpen” and “posClosed” are the actuator positions corresponding to the open/closed position.
• “transitDelay_ms” is the delay in ms that the shutter requires to fully open/close. This value is not used by the Arduino controller, but simply stored and can be returned upon request to implement wait times in a control sequence. This should be measured experimentally for each shutter.
• “Label” is the label displayed on the display. By default, this is limited to 7 characters (to fit on the touch screen display) but this can be extended in the configuration file.

These shutter parameters are stored in the EEPROM of the Arduino to retain their values after an Arduino reset.

#### 2.3.7 Arduino control modules and sequence

The shutter control in the Arduino is split into modules, which can be utilized independently: the actuator module, serial communication module, the display module, the control input module, and the idle check module. In the Arduino main loop, these modules are repeatedly queried for change requests.

The optional *serial communication module* handles serial communication between the Arduino and a computer through a USB connection.

For the optional *display module*, the LCD or TFT module can be utilized. Either will display the status of the shutter and let the user change it. A debounce mechanism is included for either device to avoid accidental multiple button presses. After a period of inactivity, either screen can dim and can be brightened again by any touch (for the TFT) or button press (for the LCD).

In the *control input module*, the control input lines are mapped to the Arduino’s pin change interrupt mechanism. Even though interrupts can suspend all other Arduino activity when called, we decided to simply queue the state changes to be handled in the main loop. Given the relatively slow mechanical response time of an RC servo, the much more involved handling within the interrupt routine would not provide a noticeably improved response time. As in the display module, a debounce mechanism is included to avoid rapid erroneous state change requests (for example with a mechanical switch).

The *idle check module* (only useful in the servo motor configuration) checks when the controller last received a state change request and disengages the servo motors if an idle time has been exceeded. This can allow the user to move the shutter positions manually, which is only possible when the servos are disengaged. We found this capability to be convenient especially during optics alignment, where the shutter controller always seemed to be just out of easy reach. This option is not applicable to the solenoid configuration since the spring closes the shutter without drive current.

#### 2.3.8 Library

We provide a library for serial communication with the Arduino in both C and Python. The provided functions in the libraries handle the low-level serial communication and provide easy-to-use access functions. Both libraries utilize the Virtual Instrument Software Architecture (VISA) standard and provide wrapper functions (e.g. to set the shutter parameters or to open/close the shutters).

In addition to these libraries, we provide an example graphical user interface (GUI) in python, based on the tkinter library. Even though NI LabWindows/CVI is a commercial program (not freely available), it is a C IDE that is used in many labs (including ours) and as a convenience we also provide the source to build a GUI using this platform.

## 3 Design files summary

**Table:** 

Design file directory name	File type	Open source license	Location of the file
Actuator Mounts	CAD	*CERN-OHL-W-2.0*	Zenodo
Enclosures	CAD	*CERN-OHL-W-2.0*	Zenodo
Arduino Code	Source	*CERN-OHL-W-2.0*	Zenodo
C Library	Source	*CERN-OHL-W-2.0*	Zenodo
C GUI	Source	*CERN-OHL-W-2.0*	Zenodo
Python Library	Source	*CERN-OHL-W-2.0*	Zenodo
Python GUI	Source	*CERN-OHL-W-2.0*	Zenodo

The repositories (Zenodo and GitHub) contain all required design files.

The *Actuator Mounts* directory contains CAD files (both STEP and STL files) to 3D print mounts for attaching the RC servos and solenoids to an optical post. Included files: ServoPostMount, ServoPostMount_Small, SolenoidPostMount.

The *Enclosures* directory contains CAD files (both STEP and STL files) to 3D print an enclosure for the shutter controller. Included files: Enclosure_Bottom, Enclosure_Top_TFT, Enclosure_Top_LCD, Enclosure_Buttons_LCD.

The *Arduino Code* directory contains the C source code for the Arduino shutter controller. Included files: ShutterDriverUniversal.ino, Common.h, RCServo.cpp, Solenoid.cpp, SerialComm.cpp, LCD.cpp, TFT.cpp, Parameters.cpp, DigInput.cpp, and associated header files.

The *C Library* directory contains source files for the C library that handles communication with the shutter controller. Included files: ArdShutter.c, ArdShutter.h.

The *C GUI* directory contains source and resource files to build a test GUI to configure and test the shutter controller. Depends on the C library above and needs NI LabWindows/CVI to be installed. Included files: ArdShutterTest.c, ArdShutterTest.h, ArdShutterTest.uir.

The *Python Library* directory contains source files for the python library that handles communication with the shutter controller. The logging level can be adjusted to include informational messages for debugging. Included files: ard_shutter.py.

The *Python GUI* directory contains source files to build a test GUI to configure and test the shutter controller. Depends on the python library above. Included files: ard_shutter_test.py, ard_shutter_panel.py.

## 4 Bill of materials summary

**Table:** 

Designator	Component	Number	Cost per unit −currency	Source of materials	Material type
*Controller*	*Arduino Uno*	1	$27.60	Arduino.cc	Semiconductor
*Controller*	Servo shield	0 or 1 *	$17.50	Adafruit	Semiconductor
*Controller*	Motor Shield	0 or 1 *	$19.95	Adafruit	Semiconductor
*Controller*	LCD shield	0 or 1	$19.95	Adafruit	Semiconductor
*Controller*	TFT shield	0 or 1	$44.95	Adafruit	Semiconductor
*Controller*	Arduino power supply	0 or 1	$8.95	Adafruit	Semiconductor
*Controller*	Shield power supply	1	$14.95	Adafruit	Semiconductor
Controller	Various electronics		∼$10	Adafruit	Semiconductor
Shutter	Actuator (servo or solenoid)	>1	∼$5–$35	Varies	Semiconductor
Shutter	Various screws, metal pieces		∼$10	Varies	Metal

Note: At least one of * is required.

*Cost analysis:*

To assemble a single-shutter system, we need an Arduino (∼$30), an Arduino power supply (∼$10, if not running off USB power), one of the shields (∼$20), a shield power supply (∼$15), an actuator (∼$10, servo or solenoid), and ∼$20 in other costs (screws, cable, connectors, etc.), totaling about $100, or about $120 if we add an LCD shield. Adding 3 more actuators to make it a four-shutter system only adds about $20 for the actuators, totaling about $140 (with display).

As a reference, we summarize approximate prices for some shutter systems that include a shutter and a driver that allows for control via a computer and a digital control signal:

• Our DIY single-shutter system: $120. Four-shutter system: $140.
• Single-shutter system, Vincent Associates (FS + VLM1 Value Pack, 25 mm mounted shutter with a single-channel driver): $865. Scale to four-shutter system: $3,460.
• Single-shutter system, Thorlabs (SHB05, 1/2″ diaphragm shutter with single-channel driver): $1,030. Scale to four-shutter system: $4,120.
• Single-shutter system, EOPC (SH-10, 13 mm rotary solenoid shutter with DSH-10 single-channel driver): $610. Scale to four-shutter system: $2,440.
• Single-shutter system, Radiant dyes (Mini Servo Motor ($215) with 4-channel controller ($485)): $700. Four-shutter system (4 servos, same controller): $1,345.
• For completeness, the commercial shutter we used as a reference for testing (Vincent Associates VS14S2ZM1) costs $1,130 and the 4-channel high-performance driver (Vincent Associates VMM-D4) costs $3,000 (not including the additional shutters).

*Sustainability and scalability:*

The parts used in the construction of this shutter system are widely available, often as generic replacements at even lower prices from other sources (for example, clones of the Arduino Uno). Servo and motor drivers are commonly used in robotics, as are servos and solenoids. In addition, the design of the shutter system is not specific to a particular model of actuator; hence, repair or upgrading to a different model does not require a re-design.

The current design can accommodate up to 16 servo shutters (with an LCD display) and 4 solenoid shutters (with either display) on the same controller. Scaling the system up to a higher number of shutters is straightforward, though a few constraints need to be kept in mind:

• One servo shield can control up to 16 servo motors. Additional shields (in principle up to 62) can be stacked and used with only minor code changes.
• One motor shield can control up to 4 solenoids. Additional shields (in principle up to 32) can be stacked and used with only minor code changes.
• Should several servo/motor shields be stacked, the case will have to be enlarged to accommodate the higher stack.
• The power supply for the actuators needs to be scaled up with the number of devices.
• The LCD display can already accommodate any number of shutters, for ease of touching the correct buttons the TFT is currently programmed for a maximum of 4 shutters. More shutters on the TFT display would require smaller fonts/buttons or a provision for scrolling.

## 5 Build instructions

General safety notice:

The assembly of the shutters and shutter controller involves 3D printing, mechanical assembly, electrical wiring, and soldering. To prevent damage to the electrical components, test power supply voltages before wiring cables to the Arduino and/or shield. All usual safety precautions should be taken when working with electronics or while soldering.

### 5.1 Shutter

The actuator mounts (for the servo motors and solenoids) were designed in a commercial CAD program (but we include the files in the universal STEP format), converted to a 3d printing format using Ultimaker Cura, and printed on a 3d printer (Creality Ender 5 Pro) with black PLA filament on raft base using the “Standard Quality” slicer parameters (layer height: 0.2 mm, infill density: 20 %, wall thickness: 0.8 mm).

#### 5.1.1 Servo motor mount

**Fig. 3:**
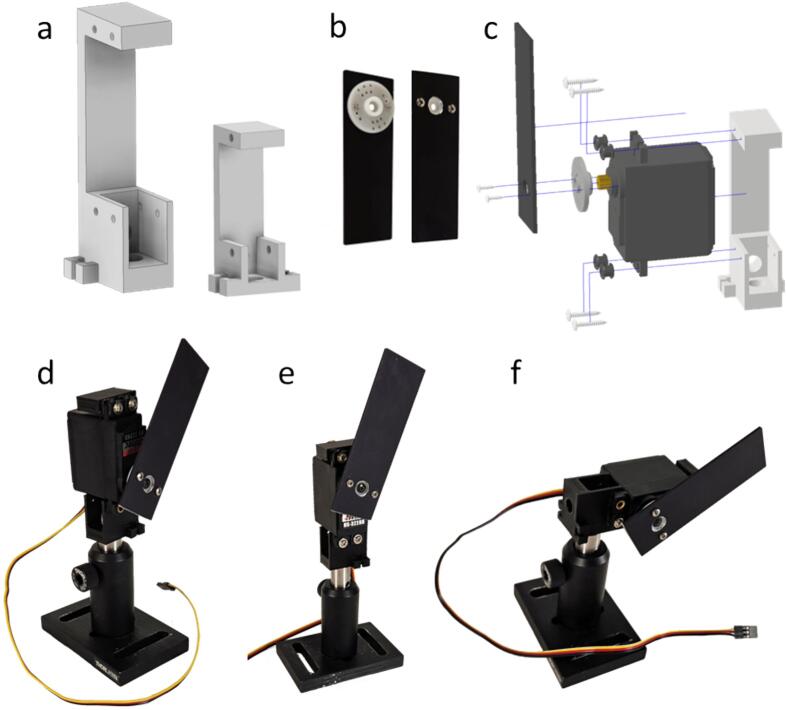
Mounting of the servo shutter: Small and large servo mounts (a), back and front view of the mounted blade (b), exploded view of the shutter assembly with the large servo mount (c), assembled shutters mounted vertically (axis down (d) or up (e)) or horizontally (f). CAD design for the servo: [28].

Two sizes of mounts (small and large) are provided for two common servo sizes (Fig. 3a). Small tabs are provided to fix the servo cables, if desired. For the blade, we drill a central hole large enough to clear the ledge on the mounting horn, and two small holes to attach it to the horn with self-tapping screws (Fig. 3b). Assembly steps are indicated in the exploded view in Fig. 3c: The servo slides in the U-shaped opening in the mount and is secured by four self-tapping screws (usually provided with the servo). Depending on the servo, rubber grommets are provided to minimize vibrations transmitted from the servo to the mount. The servo can be mounted with its axle near the post mounting screw holes (Fig. 3d) or opposite (Fig. 3e), depending on the required clearance for the light beam. The assembled servo shutter can be fixed with a socket head screw (8–32 or M4 for the small mount, ¼-20 or M6 for the large mount) to a post, vertically (Fig. 3d,e) or horizontally (Fig. 3f), depending on the light path requirements.

#### 5.1.2 Solenoid mount

**Fig. 4:**
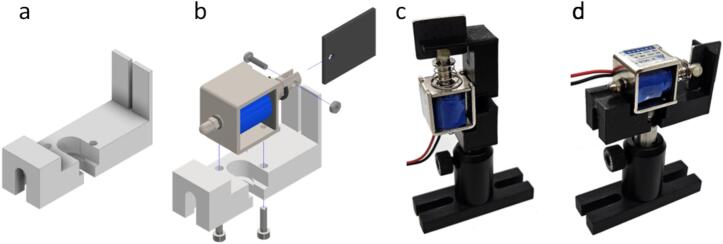
Mounting of the solenoid shutter: Solenoid mount (a), exploded view of the shutter assembly (b), and assembled shutter mounted vertically (c) or horizontally (d). CAD design for the solenoid: [29].

The solenoid mount (Fig. 4a) was designed for a specific but widely available solenoid (JF-0826B). Assembly steps are indicated in the exploded view in Fig. 4b: The solenoid is held in place by two M3 screws. The shutter blade is attached to the solenoid plunger by a plastic screw. The two guiding tabs on the mount prevent the blade from rotating (guiding is required since the non-keyed plunger can rotate). The assembled solenoid shutter can be fixed with a socket head screw (¼-20 or M6) to a post, vertically (Fig. 4c) or horizontally (Fig. 4d), depending on the light path requirements.

### 5.2 Controller hardware

#### 5.2.1 Arduino

**Fig. 5:**
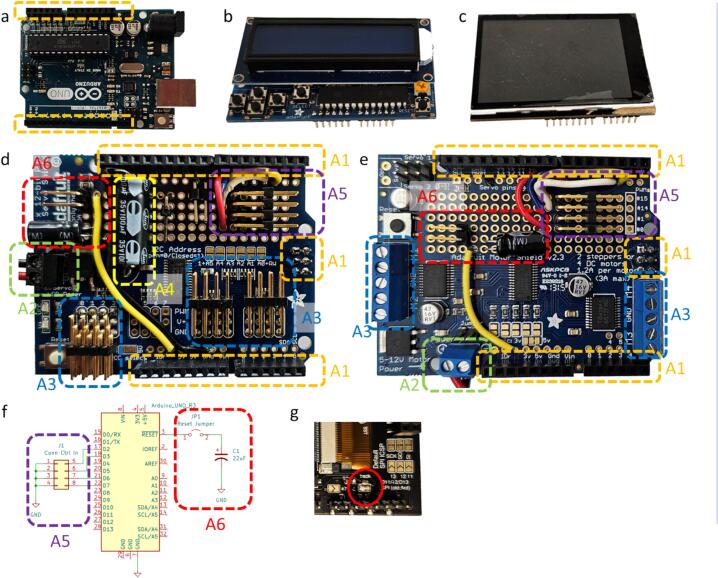
Electronic modules: Arduino (a), LCD shield (b), TFT shield (c), servo shield (d), and motor shield (e). Areas on the shields marked with color are: headers (A1), shield power connector (A2), actuator connectors (A3), servo power supply capacitor (A4), right-angle control input connector (A5), and reset bypass capacitor and jumper (A6). Simplified electrical schematic (f), indicating electrical hookup in areas A5 and A6. Close-up of the dimming solder pad on the TFT shield (g).

To mount the servo shield, a set of female headers needs to be soldered into the two rows of pins (Fig. 5a).

#### 5.2.2 Servo shield

Technical details for this shield are provided by Adafruit [30]; an assembled shield is shown in Fig. 5d. To accommodate the display shield, two rows of female through headers and the upside-down SPI female through header need to be soldered into the servo shield (Fig. 5d, A1). A 2-pin screw terminal is soldered onto the shield for the power supply cable (A2), while the other end of the cable is attached to a power supply connector. The use of a cable connector reduces the risk of accidentally plugging the servo power supply into the Arduino power connector and vice versa (both power supplies have the same connector but a different voltage). Leaving all solder pads for the I2C address open yields a default address of 0x40. Because the display shield sits on top of the servo shield, use of angled servo connectors is necessary (A3), unless the cables are soldered directly into the board. We face the first connector outward for ease of access. The remainder of the connectors (if installed) need to face inwards because of the installed headers. Note that installing the header in the opposite direction reverses the order of the servo connector pins (GND on the top vs GND on the bottom). A capacitor to provide surge current for the servos can be installed on the board (A4); the value depends on the expected number of servos operated (see instructions on the Adafruit site). For the control input, we soldered another angled connector onto the board and connected one side to the microcontroller pins (PCINT18, PCINT19, PCINT22, and PCINT23 (Arduino pins D2, D3, D6, and D7; note that D4 and D5 are used by the TFT shield) and the other side to a common ground (see area A5). Finally, as shown in Fig. 5f, we connected a capacitor in series with a jumper to the reset pin of the Arduino (A6). This is a peculiarity when using the Arduino with the VISA library, where session initialization toggles the DTR line, which resets the Arduino. Connecting a capacitor between reset and ground suppresses this line toggle and allows for opening the serial port without reset [31]. During firmware programming of the Arduino, the capacitor needs to be disconnected by removing the jumper.

#### 5.2.3 Motor shield

Technical details for the shield are provided by Adafruit [32]; an assembled shield is shown in Fig. 5e. The instructions for the through-headers (A1), the power supply connector (A2), the connector for the digital control lines (A5), and the reset bypass (A6) are identical to the servo shield above. Leaving all solder pads for the I2C address open yields a default address of 0x60. The connection to the solenoids is made by screw terminals (A3); the polarity is not important for the solenoids we use.

#### 5.2.4 LCD shield

The LCD shield (Fig. 5b), if used, is the topmost shield; hence, only short male headers are required (not stackable headers). Assembly instructions are provided by Adafruit [33]. No hardware I2C address selection is required.

#### 5.2.5 TFT shield

The TFT shield (Fig. 5c), if used, is the topmost shield; hence, only short male headers are required (not stackable headers). Assembly instructions are provided by Adafruit [34]. No hardware I2C address selection is required. On the bottom side of the shield there is a solder pad for the backlight of the screen (labelled back lite #5) – if screen dimming is to be enabled, this solder pad needs to be shorted with a dab of solder (see Fig. 5g).

#### 5.2.6 Cables

The cables supplied with the actuators are likely to be too short for typical use and extension cables are required. For servo motor cables, RC servo connector kits are available from online retailers that contain extension cables, connectors, and (if required) a crimp tool for the connectors. To reduce noise pickup from the digital PWM or other transient signals by other electronics, a shielded cable (multi-conductor + braided shield) can be used instead of ribbon cables. In this case, connect the braided shielding to GND on the Arduino side of the cable. For the control inputs we use a short, stranded wire to the BNC connectors in the enclosure.

#### 5.2.7 Power supplies

The Arduino can be powered through the USB port. If no USB connection is used (e.g. as a stand-alone shutter controller) a standard 9 V, 1 A wall-mount power supply is sufficient. For the servo shield, a 5 V power supply is recommended. The voltage for the motor shield depends on the solenoid used (max 12 V) – we also use the 5 V supply for our solenoids. The current rating depends on the number and type of actuators that are being used (we use a 3 A supply for 4 actuators).

#### 5.2.8 Controller enclosure

**Fig. 6:**
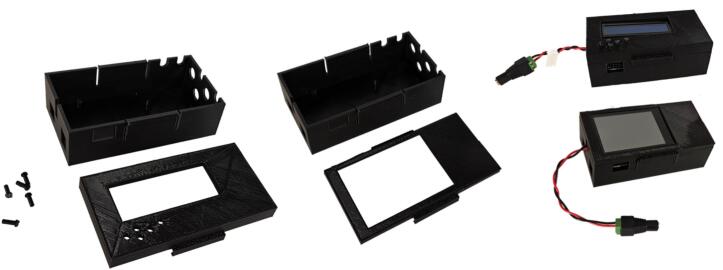
Pictures of the enclosure for a controller in parts (left) and assembled (right).

The Arduino controller (incl. shields) is enclosed in a 3D-printed box, see Fig. 6. We provide designs for controllers with one actuator (servo or solenoid) shield and an LCD or a TFT shield. The enclosure is printed in two sections that are latched together. The bottom section has standoffs and guiding pins to position the Arduino and shields, D-shaped cutouts for the BNC connectors (used for the control lines), cutouts for power supply and USB cables, a rectangular cutout for up to 4 servo connectors, and cutouts for additional actuator cables. The bottom section can be used for LCD and TFT displays, whereas the top section is specific to the display type. Both top designs have cutouts for the display; the LCD model requires the insertion of small pins for the push buttons (the flared ends are inside the box to prevent them from falling out − assembly is easiest with the enclosure turned upside down). Tabs on the outside of the enclosure are provided for mounting on an optical table.

### 5.3 Controller software

#### 5.3.1 Arduino code (firmware)

Before the shutter can be used, the Arduino code needs to be customized, compiled and uploaded. Opening the main “ShutterDriverUniversal.ino” in the Arduino IDE will open all associated source files in the same directory as well. The code is split into several modules (a C++ file with a corresponding header file each). Each module has a compiler define “SERIAL_DEBUG” that can be set to 1 to receive status and warning messages via the serial monitor for debugging purposes. For normal operation these should be set to 0. The main module (“ShutterDriverUniversal.ino”) contains the main Arduino setup and loop functions. Both functions instantiate and/or access objects/functions from the other modules. The actuator modules (“RCServo.cpp” and “Solenoid.cpp”) handle the actuator initialization and movement, the communications module (“SerialComm.cpp”) the communication over the serial (USB) port, the display modules (“LCD.cpp” and “TFT.cpp”) the display and user input, the parameters module (“Parameters.cpp”) the parameter storage in the EEPROM, and the digital control module (“DigInput.cpp”) the shutter control via the control lines.

For convenience, the customization via user-adjustable parameters is done in the file “Common.h”. In Table 3 we provide a step-by-step list of configuration options. The most important step is to (un)comment the compiler defines that indicate the modules to be included or excluded during compilation. The table lists the important parameters in “Common.h”, but less common ones (such as the width of the borders on the TFT, the servo control frequency, etc.) can be found in the file, with associated comments explaining their function.

**Table 3:** Configuration steps for the file “Common.h”.

Is serial communication required?	No: Comment SERIALCOMM define
	Yes: Uncomment SERIALCOMM define Pick the desired baud rate (SERIAL_BAUDRATE) and termination character (SERIAL_TERMCHAR − carriage return or line feed)
Which display is used? Note: only one (if any) of the two can be used	LCD: Uncomment DISPLAY_LCD and comment DISPLAY_TFT define Set the desired switch debounce time (LCD_BLOCKING_TIME_MS) in ms Set the time after which the display is dimmed (LCD_DIM_PERIOD_S) in sec (0 for no dimming)
	TFT: Uncomment DISPLAY_TFT and comment DISPLAY_LCD define Set the desired touch debounce time (TFT_BLOCKING_TIME_MS) in ms Set the time after which the display is dimmed (TFT_DIM_PERIOD_S) in sec (0 for no dimming)
What kind of actuator is used? Note: exactly one of the two must be used	Servos: Uncomment SHUTTER_RCSERVO and comment SHUTTER_SOLENOID define Set the shield I2C address (RCSERVO_BOARDID − default is set) If you want the servos to release after a given time, adjust IDLEINTERVAL_S (in sec), otherwise set to 0
	Solenoids: Uncomment SHUTTER_SOLENOID and comment SHUTTER_RCSERVO define Set the shield I2C address (SOLENOID_BOARDID − default is set) Set IDLEINTERVAL_S to 0
Are the digital control lines used?	No: Comment DIGINPUT define
	Yes: Uncomment DIGINPUT define Choose whether to use the internal pullup resistors with DIGINPUT_USEPULLUPS (1-> use pull-ups, use for switches; 0-> no pull-ups, use for TTL signals) If switch debouncing is required, adjust the DIGINPUT_CHECK_INTERVAL_MS and DIGINPUT_MAX_CHECKS parameters (see comments in file for details), otherwise set DIGINPUT_CHECK_INTERVAL_MS to 0

To compile the source code in the Arduino IDE, several libraries need to be included if the respective shield is in use (the help menu in the IDE provides a link to library install instructions): the Adafruit PWM Servo Driver library, the Adafruit Motor Shield V2 library, the Adafruit RGB LCD Shield library, and the Adafruit FT6206 library (for the TFT shield). Make sure to allow installation of dependent libraries by the Arduino IDE. After successful compilation, the Arduino code needs to be uploaded via the IDE (if the reset capacitor was installed, make sure to remove the jumper for uploading).

#### 5.3.2 C library

The C library provides helper functions to assist communication of a computer with the Arduino shutter controller (e.g. to set the shutter parameters or to open/close the shutters). The library handles low-level serial communication and provides easy-to-use wrapper functions. It utilizes the Virtual Instrument Software Architecture (VISA) standard, which needs to be installed on the computer. Free VISA implementations (with installation instructions) are available from several companies, such as Tektronix [35], Keysight [36], or Rohde & Schwarz [37]. The supplied code was tested with NI-VISA [38] (which is, as of the time of writing, no longer free). The shutter C library (ArdShutter.c) only depends on the VISA library. The serial baud rate and termination character are defined in the same file. The header file (ArdShutter.h) only contains function prototypes for inclusion in other modules. The library currently only supports a single Arduino shutter controller and VISA handles are stored internally in the module. An instrument session needs to be established with ARD_ShutterInit before shutter commands can be issued. The session needs to be closed with ARD_Close when finished.

#### 5.3.3 Python library

The python library provides the same functionality as the C library above. This library, too, utilizes the Virtual Instrument Software Architecture (VISA) standard. For this library to work, the pyVISA library [39] needs to be installed. While pyVISA can use an installed VISA library from the beforementioned sources, it can also utilize pyVISA-py [40], an open-source, python-based VISA implementation. The supplied code was tested with pyVISA and pyVISA-py. The shutter controller is implemented as a class; its constructors and destructors handle instrument initialization and closing.

#### 5.3.4 C GUI

The C GUI tests the shutter functionality and uses the above C shutter library. To be compatible with the rest of our lab software, we used NI LabWindows/CVI (not free or open source − see the python GUI below for an open-source implementation) for incorporation of the shutter into our experiment. Though the source will not compile without the NI suite, the source can serve as example code on how to use the C shutter library functions.

#### 5.3.5 Python GUI

The python GUI tests the shutter functionality and uses the above python shutter library. The GUI is based on Tkinter, which comes built-in with most python installers.

## 6 Operation instructions

General safety notice:

The shutters are designed to block light impinging on the shutter blade. Some heating of the blade and light scattering off the blade is expected and needs to be managed and monitored. When setting up and calibrating the shutters, adhere to all light (or laser) safety precautions.

### 6.1 Initial setup

Before the shutters attached to the controller can be used, the parameter settings need to be determined and uploaded to the controller. Upon boot, the Arduino reads saved parameter values from EEPROM, but does not move any actuators unless directed by commands or user input. This gives the user the chance to safely program the parameters before first use (the EEPROM could initially contain random values). The following is the sequence for initial use (can be performed using the library functions, with one of the GUIs, or directly with serial commands via the Arduino serial monitor):

1. Unplug the Arduino.
2. Connect the shutters to the servo or motor shield and make a note of the shield port number used.
3. Plug the Arduino into a serial port.
4. Adjust the header file “Common.h” (see Table3), compile, and download the Arduino code with the Arduino IDE.
5. Clear the parameter setting with the CLR command (see “Notes” in case of a stand-alone shutter controller).
6. For each shutter, send parameter values with the SPR command using “-1” as the shutter number – this value adds a new shutter to the parameter list. Make sure the port numbers match the ones from step 2. For servos, use default values (e.g. 100,200,300) for the parameters openPos, closePos, and transitDelay (these values are calibrated in the next step). For solenoids, use 0 for closePos and 255 for openPos.
7. Place the shutters in the respective beam path.
8. In case of servo motors: For each shutter, adjust the RC servo positions directly with the SSP command to find appropriate open and closed positions (note that if a position is unreachable, the servo horn can be attached at a different angle). Update the openPos and closePos parameters for each servo with the SPR command, this time using the respective shutter number (instead of the −1 used previously, remember that the index is zero-based). An easy way to do this is to get the parameters with the GPR command, change the positional values, and send the updated parameters back with the SPR command.
9. If the transit delay parameters are used (these do not affect the shutter operation and are just stored for queries) they need to be calibrated, for example with a photodiode in the light path. Once determined, update this value (like the update in step 8).
10. Check the parameter values (and order) individually.
11. Save the parameters to EEPROM with the SAV command.

Notes:

a) If no serial connection is used in the shutter controller, the parameters can be set when downloading the program to the Arduino (see Note b below).
b) In the unlikely event that random initial values in the EEPROM make the Arduino behave erroneously when first programmed and powered up (or when no serial connection is used for the shutter operation), we provide a routine “createDummyParameters” in the file “ShutterDriverUniversal.ino” that could be temporarily substituted for “_params.readFromEEPROM” in the setup portion of the Arduino to pre-set the parameters in the EEPROM (see instructions in the source code).

### 6.2 Standard operation

The shutter parameters are stored in the Arduino and are loaded at boot time. No calibration is needed during normal operation. The following features are enabled by default (but can be disabled in the source code, see Table 3):

• The screen dims after a period of inactivity (default 60 s, set LCD_DIM_PERIOD_S or TFT_DIM_PERIOD_S to zero to disable). Wake-up is through any button press or touch.
• For the LCD display, the up/down buttons cycle through the shutters, the left/right closes/opens them. For the TFT display, actions are button touches.
• The RC servos can be made to disengage (after which they are movable by hand) after a period of inactivity (default 5 s, set IDLEINTERVAL_S to zero to disable this mode). This only applies to servos, not to solenoids.

## 7 Validation and characterization

### 7.1 Test setup

**Fig. 7:**
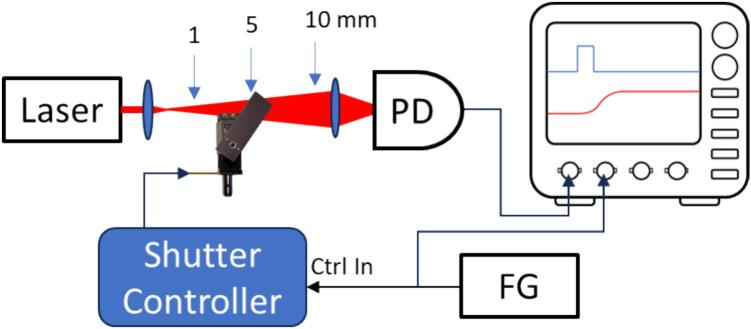
Test setup to measure opening, closing, and delay times. PD: photodiode, FG: function generator.

The performance of the shutter (opening/closing times and delays) varies widely with use parameters, like the actuator type, size of the blade, size of the light path, and relative positioning. For testing in the setup shown in Fig. 7, we used a laser beam from a laser diode (Thorlabs PL252) and expanded the beam with a lens (we tested the shutter at beam diameters of 1 mm, 5 mm, and 10 mm). After the shutter position, the laser was focused onto a photodiode and its output was monitored with an oscilloscope. A TTL pulse from a function generator toggled the shutter (open/close) through the control input of the shutter controller and provided a reference for the oscilloscope. With this setup, we measured the opening/closing and delay times. We define the opening time as the rise in photodiode signal from 20% to 80% full scale and the closing time as the fall time from 80% to 20%. The opening/closing delay is the time from the change in the control signal to the midpoint of the opening/closing signal.

Several shutter configurations were measured:

• **“Large Servo”** − a servo motor shutter with a large-frame servo (HiTEC HS-322HD). In this configuration, the shutter blade pivot point was about 5.5 cm from the beam.
• **“Small Servo”** − a servo motor shutter with a small-frame servo (Savox SH-0262MG). In this configuration, the shutter blade pivot point was about 3 cm from the beam.
• **“Solenoid (horizontal)”** − a solenoid shutter with a solenoid (JF-0826B), mounted horizontally. This solenoid has a theoretical throw of 10 mm, but a rubber gasket for damping reduces the throw to about 8 mm, hence the 10 mm diameter beam could not be measured.
• **“Solenoid (vertical)”** – same solenoid as above but orientated vertically.
• **“Commercial”** A commercial shutter system (Vincent Associates shutter VS14S2ZM1 and a VMM-D4 driver).

In all cases, the shutter was positioned such that the beam was at least 2 mm from the edge of the blade (in the open and closed positions). For each configuration, 100 opening/closing cycles were measured.

### 7.2 Results

**Fig. 8:**
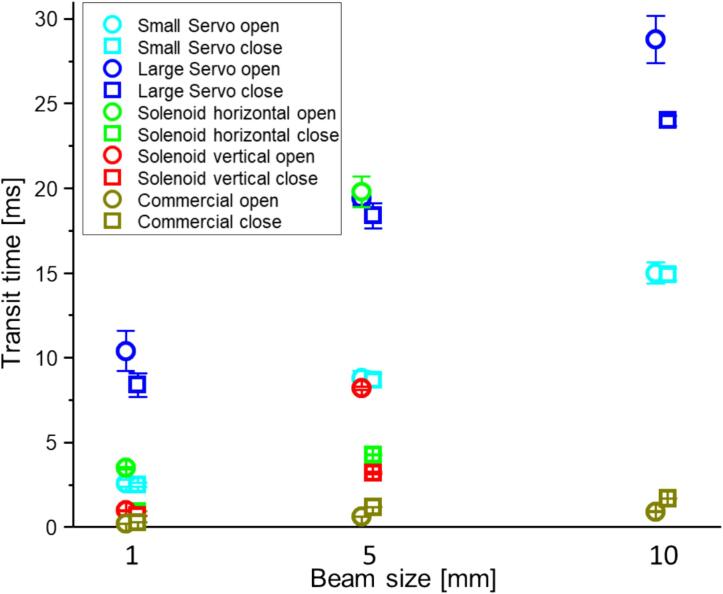
Measured opening and closing times τ (values from Table 4).

**Fig. 9:**
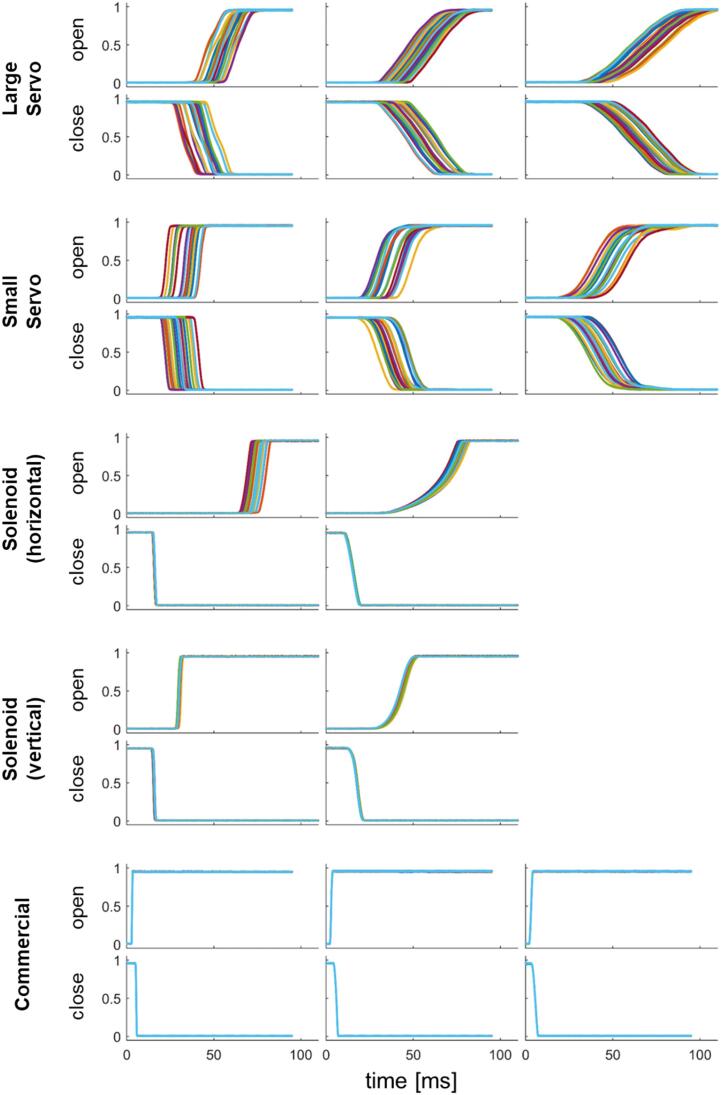
Measured beam transmission during opening and closing of various shutters. The time t = 0 corresponds to the rising/falling edge of the digital control signal. 20 repetitions are shown each.

Table4 shows the experimental opening and closing transit times τ and delays T (averages and sample standard deviations). The transit times τ are also plotted in Fig. 8. Finally, Fig. 9 shows photodiode traces for these configurations (only the first 20 iterations are shown for clarity).

**Table 4:** Measured opening and closing times (τ) and delays (T).

Shutter type	Direc-tion	τ [ms] (∅1 mm)	T [ms] (∅1 mm)	τ [ms] (∅5 mm)	T [ms] (∅5 mm)	τ [ms] (∅10 mm)	T [ms] (∅10 mm)
Large Servo	open	10.4 ± 1.2	55 ± 5.5	19 ± 0.37	55 ± 5.5	29 ± 1.4	70 ± 5.9
	close	8.4 ± 0.71	42 ± 5.7	18 ± 0.75	55 ± 5.5	24 ± 0.3	67 ± 5.5
Small Servo	open	2.6 ± 0.19	32 ± 5.6	8.8 ± 0.44	39 ± 5.5	15 ± 0.63	48 ± 5.8
	close	2.5 ± 0.12	30 ± 5.7	8.7 ± 0.33	40 ± 5.5	15 ± 0.36	45 ± 5.4
Solenoid (horizontal)	open	3.5 ± 0.080	74 ± 3.2	20 ± 0.92	70 ± 2.5	−	−
	close	0.95 ± 0.003	16 ± 0.18	4.3 ± 0.015	15 ± 0.17	−	−
Solenoid (vertical)	open	1.00 ± 0.019	30 ± 0.38	8.2 ± 0.084	44 ± 0.8	−	−
	close	0.69 ± 0.004	16 ± 0.19	3.2 ± 0.057	18 ± 0.19	−	−
Commercial	open	0.20 ± 0.001	3.1 ± 0.004	0.63 ± 0.003	2.9 ± 0.005	0.94 ± 0.003	3.2 ± 0.006
	close	0.30 ± 0.001	5.5 ± 0.006	1.2 ± 0.003	5.9 ± 0.002	1.7 ± 0.015	5.5 ± 0.013

### 7.3 Servo shutter performance

For our servo configurations, the faster rotational rate of the smaller servo (rated speed of 60 deg/80 ms) over the larger servo (60 deg/190 ms) is partially offset by the shorter lever arm (3 cm vs. 5.5 cm). Based on Eq. (1) we would expect the small servo to be 25% faster than the large servo. We see a larger difference, which likely results from the approximations used for Eq. (1) – it assumes a uniform (top-hat) beam (our beam is not), calculates 0 to 100% transit times (we quantify 20% to 80% because a non-top-hat beam doesn’t provide sharp cut-on and cut-off points), and neglects acceleration effects.

We expected the delay times to depend strongly on the positioning of the shutter with respect to the beam and we observed delays up to about 50 ms (the debounce of the digital control inputs was disabled). However, we also observed large shot-to-shot variations in the delay times in a range of 20 ms. This timing jitter is consistent with the 50 Hz operating frequency of the PWM controller on the servo shield. Any update request to the PWM controller that occurs between the pulse repetition period (20 ms) is deferred until the next period. Hence, relative to the digital control signal the servo movement starts anywhere within a 20 ms window. Direct control of the PWM waveforms (via the internal Arduino timers) might improve the performance but given the programmatic complexity we did not attempt this.

### 7.4 Solenoid shutter performance

While servo motors have a fairly well-defined rotational velocity profile, the movement of our solenoids depends much more strongly on external parameters, such as the applied voltage, the mass of the plunger, friction, etc. In our experiments, we noticed that the transit time and the delay depend strongly on the orientation of the shutter. In the horizontal configuration, the solenoid movement is slower with a somewhat larger variation, especially during opening. When mounted horizontally, the blade experiences stronger friction on the mount, while in the vertical orientation the blade is just loosely guided and experiences very little friction. In addition, when mounted vertically, gravity helps pull the plunger down during opening, which could help shorten the opening time. In the vertical configuration, the solenoid shutter is as fast as (or faster than) the servo-based shutter with much less timing jitter. The drawback, however, is that the solenoid causes more vibrations and a louder noise than the servo-based design. Mounting with damping material (such as sorbothane) could provide some improvement in this regard.

### 7.5 Commercial shutter performance

We compare the commercial shutter performance for our largest measured beam (10 mm diameter) to the shutter’s specification for a beam filling its aperture (14 mm). This shutter exhibits short transit times and very stable timing. The opening time for the 10 mm beam was about 1 ms and the closing time 1.7 ms, which are within the shutter’s specifications of 1.5 ms and 3 ms, respectively. The opening delay time of 3.2 ms is also within the specified signal-to-80%-open time of 3.5 ms (the closing delay is not specified). The repeatability of the shutter is also not specified, but from our measurement we obtain an excellent performance with a timing jitter of no more than 20 µs.

### 7.6 Performance summary and possible improvements

The performance tests show that our DIY shutter system cannot (and was not meant to) compete with commercial shutters in terms of speed and precision. However, despite the lower speed we believe that our design will find uses in many areas such as laser science, spectroscopy, and microscopy, especially when multiple shutters are required. Our design is open source, easy to assemble, and costs much less than commercial devices, greatly aiding its potential for widespread use.

In future work will explore the use of rotary solenoids. Though more expensive than linear solenoids, they likely offer advantages in speed and repeatability (lower mass of the moving parts, less friction effects). Some commercial shutters, such as [10], [11], [12], [13], already use rotational solenoids and we expect that our controller design will work with these devices with only minimal (if any) adjustments.

### CRediT authorship contribution statement

**Mathias S. Fischer:** Writing – review & editing, Validation, Software, Investigation. **Martin C. Fischer:** Writing – original draft, Supervision, Software, Methodology, Funding acquisition, Conceptualization.

## Declaration of competing interest

The authors declare the following financial interests/personal relationships which may be considered as potential competing interests: Martin Fischer reports financial support was provided by National Science Foundation. Martin Fischer reports financial support was provided by The Chan Zuckerberg Initiative. If there are other authors, they declare that they have no known competing financial interests or personal relationships that could have appeared to influence the work reported in this paper.

## Figures and Tables

**Table:** 

Component	Number	Cost /unit	Source	Part number	Link	Comments
Arduino Uno R3	1	$27.60	Arduino.cc	Arduino A000066	https://store-usa.arduino.cc/products/arduino-uno-rev3	Arduino clones can be obtained (cheaper) from a variety of online sources
Servo Shield	1	$17.50	Adafruit	Adafruit 1411	https://www.adafruit.com/product/1411	
Motor Shield	1	$19.95	Adafruit	Adafruit 1438	https://www.adafruit.com/product/1438	
Stacking headers	1	$1.95	Adafruit	Adafruit 85	https://www.adafruit.com/product/85	Only required if a display shield is used
Pack of right-angle headers	1	$2.95	Adafruit	Adafruit 816	https://www.adafruit.com/product/816	Only required if a display shield is used
LCD shield	1	$19.95	Adafruit	Adafruit 772	https://www.adafruit.com/product/772	Optional
TFT shield	1	$44.95	Adafruit	Adafruit 1947	https://www.adafruit.com/product/1947	Optional
9 V power supply for Arduino	1	$8.95	Adafruit	Adafruit 63	https://www.adafruit.com/product/63	Only required if not connected to USB
5 V power supply for servo shield	1	$14.95	Adafruit	Adafruit 1466	https://www.adafruit.com/product/1466	Amperage depends on the number of servos
Power adapter jack to screw terminal	1	$2	Adafruit	Adafruit 368	https://www.adafruit.com/product/368	Used to connect the servo shield power supply to the servo shield board
BNC connectors	0–4	$3.76	Digikey and others	Amphenol 31–10-RFX	https://www.digikey.com/en/products/detail/amphenol-rf/31-10-RFX/100642	Only required if control lines are used

## Appendix A BOM details

Controller

The following items might be required, but these are common items in any electronics bench:

• Electrolytic capacitor (value depends on number of servos) − only required if several servos are used (see manuscript).
• Electrolytic capacitor (∼22 µF) and a jumper − only required if serial communication resets Arduino upon port open (see manuscript).
• Some wire and solder accessories.

**Shutter**

A variety of actuators are available, the ones we tested were:

• Small servo: Savox SH-0262MG. As of the time of manuscript submission, this item is discontinued. A replacement item is SH-0264MG at a price of $35.99 each. https://www.savoxusa.com/products/savsh0264mg-super-torque-metal-gear-micro
• Large servo: MG 996R at a price of about $5 each. 4-pack: https://www.amazon.com/4-Pack-MG996R-Torque-Digital-Helicopter/dp/B07MFK266B/
• Large servo: HiTEC HS-322HD at a price of about $15 each. https://www.amazon.com/Hitec-33322S-HS-322HD-Standard-Karbonite/dp/B0006O3XEA/
• Solenoid: Medium Push-Pull Solenoid, JF-0826B, 5 V, available for $7.50 each. https://www.adafruit.com/product/3992

**Other parts:**

• If the cables of the servos are too short (very likely), extension cables can be made with a servo cable kit. A crimp tool makes assembly easier − this tool is included in many kits, for example: https://www.amazon.com/Female-Connector-Crimping-Compatible-Spektrum/dp/B0BGX7157Y/. Price: about $24 each
• For the blade, a scrap piece of anodized aluminum will work. We used a small section off a 1/8″ thick, 1 ft strip: https://www.mcmaster.com/7083T11/ Price: $12.50/ft
• To attach the blade to the servo horn, most short #0 self-tapping screw will do, e.g. https://www.mcmaster.com/92470A018/ (Price: $7/pack of 50 screws).
